# Advances in eggshell membrane separation and solubilization technologies

**DOI:** 10.3389/fvets.2023.1116126

**Published:** 2023-03-16

**Authors:** Chunhao Han, Yifan Chen, Lei Shi, Hui Chen, Lanhui Li, Zhonghua Ning, Dan Zeng, Dehe Wang

**Affiliations:** ^1^College of Animal Science and Technology, Hebei Agricultural University, Baoding, China; ^2^College of Animal Science and Technology, China Agricultural University, Beijing, China; ^3^Hebei Layer Industry Technology Research Institute, Handan, China

**Keywords:** chicken, eggshell membrane, protein, disulfide bond, dissolution

## Abstract

Eggshell membranes (ESM) contain 90% protein, 3% lipids, 2% sugars, and small amounts of minerals such as calcium and magnesium. Of the 90% of proteins present, 472 proteins species have been identified. ESM provide the initial mineralization platform for eggshell formation, and can be used for to produce adsorbents, cosmetics, and medical products because of their special physical structure and chemical composition. The special physical structure of the eggshell membrane, with disulfide bonds between and within the protein molecules and the cross-linking of lysine-derived and heterochain chains between the eggshell membrane, makes the membrane very difficult to dissolve, with a maximum solubility rate of only 62%. Also, the insolubility of ESM limits its development and use also any related research. Based on the physical structure and chemical composition of the eggshell membrane, this paper reviews the latest research on eggshell membrane separation and membrane protein solubilization to provide a reference for promoting the separation, dissolution, and rational development and use of the avian eggshell membrane.

## 1. Introduction

The world poultry egg production in 2020 was 92.967 million tons, of which chicken eggs accounted for 93.2% (1), dominating in poultry egg products. The proportion of eggshell and eggshell membrane in the egg structure is 10–12% and 1.02%, respectively (2), suggesting that 9.531 million tons of eggshell and 883,800 tons of eggshell membrane by-products are produced yearly in the world. Eggshell (mineralized layer) contains 95% minerals (93.5% CaCO_3_) and 3.4% organic matter (3, 4), exploitation of which is less and rarely reported. The eggshell membrane acts as a semi-permeable membrane (5) wrapping the egg white and preventing its exudation and external pathogens from entering the egg and safeguarding egg quality (6, 7). In addition, the eggshell membrane provides the initial mineralization platform for to form the eggshell (8) and improves the mechanical properties of the egg. By physical structure, eggshell membranes consist of a stack of membrane fibers, which have certain adsorption properties because of their porosity (9), and can be used for the adsorption and recovery of heavy metal ions in waste liquids. Due to their biocompatibility, ESM can be used as dressings for burned tissues in the medical field (10). For chemical composition, 472 proteins have been identified in ESM (11), with water-soluble collagen being the most plentiful (10%) and can be used in to produce moisturizing cosmetics; the high keratin content of ectodermal cells can be used in to produce wound healing agents and skin creams (12). The current separation methods for ESM mainly involve washing, drying, and crushing the collected eggshells, and then treating them with physical, chemical, or biological enzymatic methods, respectively, to detach the ESM from the true shells, and then sorting and recovering them, so as to achieve the purpose of shell-membrane separation.

The effect of shell membrane separation is better, and the highest recovery rate of eggshell membrane is reported to be up to 98.9% (13). However, the solubility of the eggshell membrane protein is low, practically insoluble in water and difficult to dissolve in many solvents. The highest solubility of the eggshell membrane was reported to be 62% (14). This may be due to the binding between soluble and insoluble proteins and the highly cross-linked structure formed by the intertwining of the eggshell membrane fibers.

Although shell membrane separation methods and shell membrane proteolysis methods are classified in the same way, the content of the categories and purpose of the methods used are not the same. The purpose of shell membrane separation is only to separate the eggshell from the eggshell membrane without destroying the internal structure of either. Shell membrane lysis methods increase eggshell membrane lysis in various ways so that the various proteins, fats, sugars, and other parts that make up the eggshell membrane are freed and the internal structure of the eggshell membrane is significantly changed. In this paper, we review the separation and protein solubilization of ESM with the aim of advancing eggshell membrane solubilization, providing references for further investigation of eggshell membrane composition and structure, exploring to form eggshells, and promoting to exploit ESM.

## 2. Physical structure and chemical composition of eggshell membranes

**Figure 1:**
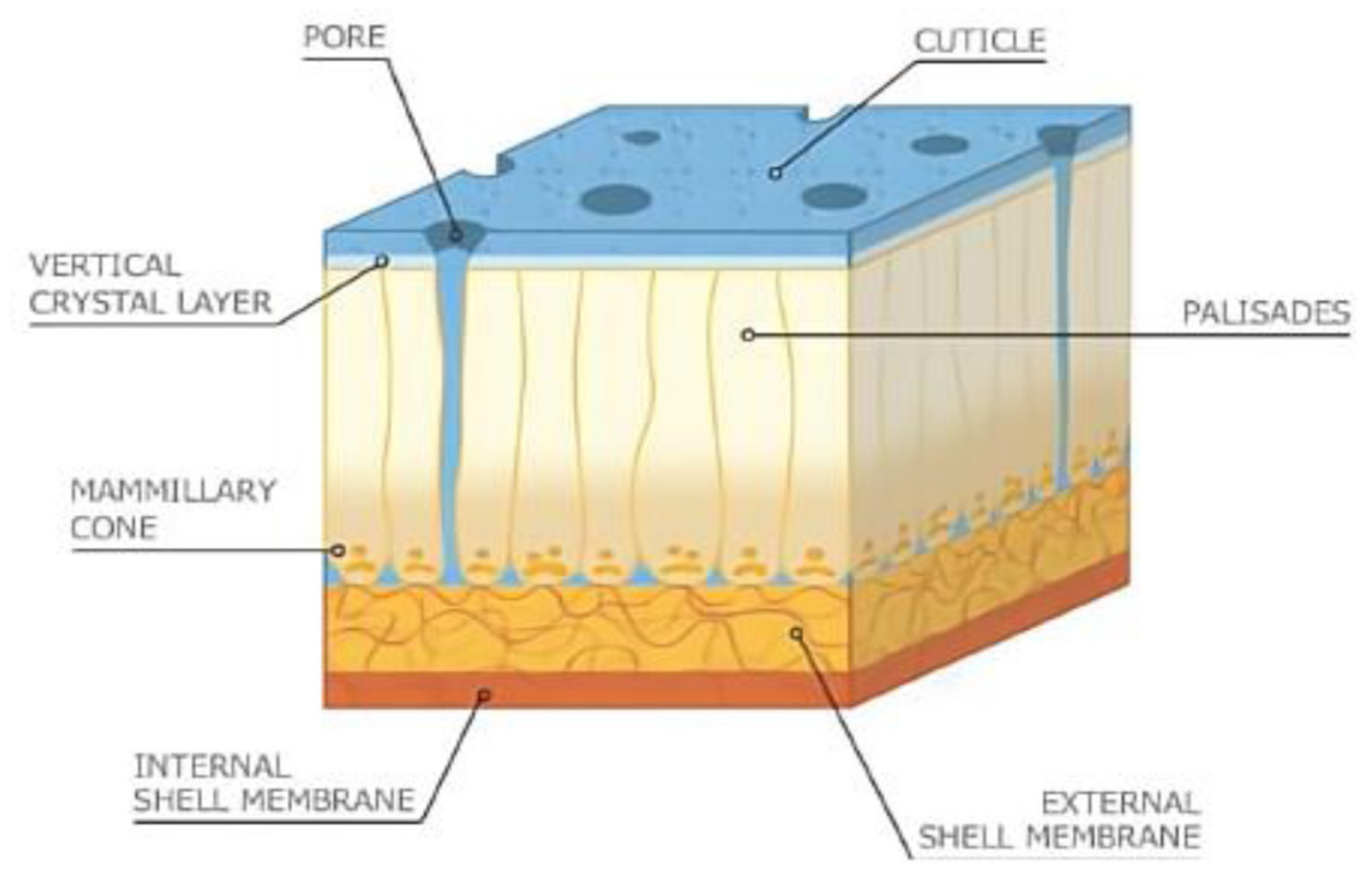
Artistic rendition of cross-sectional view of eggshell. Image reproduced from Hincke et al. (18).

**Figure 2:**
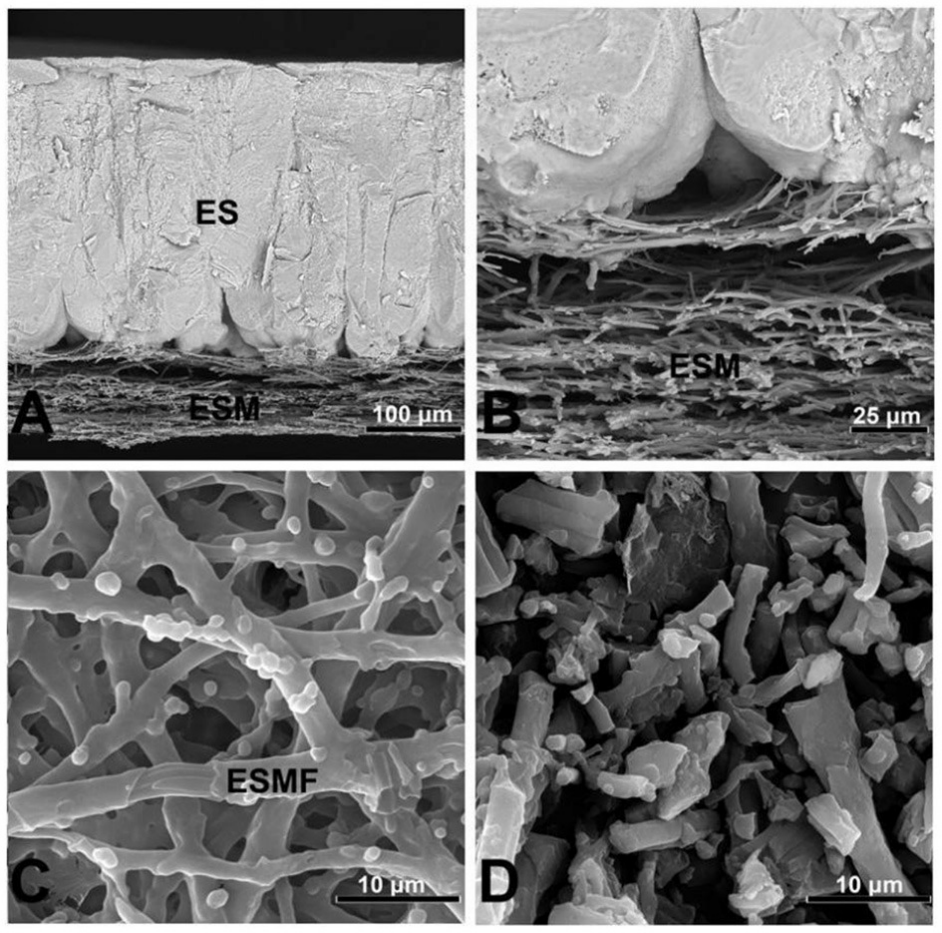
Scanning electron micrographs illustrating the morphology of the eggshell and eggshell membranes. **(A)** Eggshell cross-fractured to reveal the eggshell membranes (ESM) and calcified eggshell (ES); **(B)** higher magnification of the ESM and mammillary cone interface; **(C)** hand-peeled ESM, showing an eggshell membrane fiber (ESMF); **(D)** processed ESM, showing fragmented eggshell membrane fibers. Scale bars: **(A)**–50 μm; **(B)**–20 μm; **(C, D)**–2 μm.

The eggshell membrane is a fibrous membrane wrapped around the egg white and embedded in the eggshell papillae, structurally divided from the inside into the limiting membrane (LM), the inner eggshell membrane (ISM), and the outer eggshell membrane (OSM), with a total thickness of about 70 μm (15). Next to the outer eggshell membrane is the mineralized eggshell layer, which contains the papillae, fenestra, and vertical crystal layers (16). The inner and outer ESM consist of layers of membrane fibers about 25 μm in length (17). The artistic rendition of the eggshell is shown in Figure 1, where the internal and external ESM can be seen in the lower part (18). With the diameter of the membrane fibers gradually increasing from the inner to the outer regions (19). The summary information of the physical structure of the eggshell membrane is shown in Table 1. Chemically, the fibrils of the eggshell membrane consist mainly of proteins, of which about 10% are collagen (types I, V and X) and 70–75% are other proteins and glycoproteins containing lysine-derived crosslinks (20). In addition, the inner eggshell membrane and the outer eggshell membrane differ slightly not only in morphology but also in chemical composition. The core proteins of the inner eggshell membrane contain mainly type I and V collagen, while the outer eggshell membrane contains mainly type I ESM (21). Type X collagen was found in both membranes (22). There are 472 proteins that have been reported in ESM, including some typical proteins containing specific structures, such as lysozyme (Lysozyme), ovotransferrin, ovalbumin, and OC-17 (Ovocledidin-17). Another fraction of proteins include osteopontin and keratin, which are common protein (23). Through molecular simulation techniques, OC-17 protein has now been found to play an important role in helping the transition of CaCO_3_ from shapeless particles to calcareous crystals (24). In contrast, enzymatic digestion of ovotransferrin may provide specific benefits to human health through to prepare a large number of active peptides. For example, inhibition of bacterial and viral growth, prevention of chronic diseases, lowering of blood pressure, inhibition of tumor cell proliferation, and improvement of human immunity (25). Due to the insolubility of ESM, more membrane protein species and relative contents may need further study. Although it is difficult to measure membrane protein components due to the insolubility of ESM and protein instability, amino acids—the basic structural units of eggshell membrane proteins—can be accurately measured due to their stability in strong acids and bases. Among the 18 reported eggshell membrane protein amino acids, proline and glutamic acid are high in content, accounting for approximately 11.5–11.8% (26) and 9.15–10.28% (27), respectively. In addition, the results of the study showed that ESM are richer in cystine and cysteine (28), which may be related to the highly cross-linked structure of ESM. However, the exact relationship needs to be further verified. Scanning electron micrographs of the eggshell and eggshell membrane morphology are shown in Figure 2 (29).

**Table 1:** Summary of eggshell membrane structure characteristics.

**Structure**	**Location**	**Features**	**Thickness**	**Fiber thickness**
Outer eggshell membrane	Under the eggshell	Fibers of the outer shell membrane extend into the mammillary knobs of the eggshell	50–70 μm	1–7 μm
Inner eggshell membrane	Inner shell membrane and outer shell membrane separated by air chamber	Fibers of the inner ESM layer are interspersed with the outer membrane	15–26 μm	0.1–3 μm
Limiting membrane	A membrane surrounding the protein	The innermost thin structure	Particles filled between proteins, tiny	Granules that fill the space between inner eggshell membrane fibers

## 3. Progress in research on eggshell membrane separation technology

Since the outer ESM is embedded in the eggshell papillae, it is necessary to separate the ESM from the eggshell mineralized layer before dissolving the ESM (30). Typical eggshell membrane separation technologies are shown in Table 2. The main methods of eggshell membrane separation include physical, chemical and enzymatic methods.

**Table 2:** Typical shell membrane separation technology statistics.

**Type**	**Separate characteristics**	**Membrane recovery rate**	**References**
Physical method	By with crushing, ultrasonic irradiation treatment and sieve separation, the difference in specific gravity between eggshell membrane and eggshell is used for recovery.	85–95%	(31)
	Using pressurization and heating, the conditions are above 100°C and 1.2 Mpa,	High separation efficiency and high egg film purity	(32)
	Cyclone airflow sorting devices	94.00%	(33)
	Method and apparatus for separating egg shell and eggshell membrane	High separation efficiency and high egg film purity	(34)
Chemical method	Hydrochloric acid volume 20 mL, reaction time 19 min, hydrochloric acid concentration 0.5 mol/L	89.21%	(35)
	Separation time was 36.25 min, separation temperature of 48.96°C, hydrochloric acid concentration of 3.68 mol/L	96.52%	(36)
	3 times excess CH_3_COOH solution	93.00%	(37)
Enzymatic method	Alkaline protease and Papainp	98.90%	(38)
	50 μg/mL Proteinase K treatment for 48 h	Unclean removal of the outer egg shell membrane	(39)
	0.20 g/100 mL alkaline protease treatment for 2 h	Optimizing egg film properties	(40)

### 3.1. Physical method

The physical separation method of the eggshell membrane is mainly based on the difference of mechanical strength and specific gravity of the eggshell and eggshell membrane. The shell and membrane are mechanically crushed as a whole, and then poured into water and left to stratify, so that shell and membrane separation can be achieved. In 2020, Yuji Hasebe (31) used the difference in specific gravity between eggshell membranes and eggshells to recover batches of eggshell membranes by crushing, ultrasonic irradiation treatment and separation using a sieve, getting a recovery rate of 85–95%. They also designed a cyclone type airflow cleaning device, and the membrane recovery rate was higher than 94% and the membrane cleaning rate was higher than 96% under the optimal operating parameters (33). Another method is through dissolved air flotation (DAF) invented by using the different solubilities of water under different pressures, and about 96% of the eggshell membrane can be recovered by the DAF separation device (41). The above mechanical methods of separating shell membranes all use water as the medium. Using this method can reduce costs, save resources, and achieve a lower rate of calcium loss, but resulting acid or alkali treatment is needed to improve membrane recovery.

### 3.2. Chemical method

The chemical separation of the eggshell membrane acts on the fiber connection between the outer eggshell membrane and the eggshell mineralized papillae. Shell membrane separation was achieved by breaking the fibers between them through chemical reagents. Zhen (42) used a roller mill and then passed a 2.5 mm steel sieve to obtain 79.0% shell membrane separation in combination with the acid treatment method. Wang (36) used hydrochloric acid solution for shell membrane separation and introduced response surface analysis to optimize the separation process. By establishing a regression model analysis, it was concluded that the highest recovery of eggshell membrane was 97.81% at a hydrochloric acid concentration of 3.68 mol/L. However, this recovery was the predicted value of the recovery of eggshell membrane under ideal process conditions, and the recovery obtained from the actual test was 96.52%. The relative error between the actual value and the predicted value reached 1.29%, which proved that there was room for further optimization of the test. When using acid or alkali for eggshell membrane separation, the membrane recovery is high, but the eggshell will be dissolved and the membrane bioactivity is not easily guaranteed. Adding chemical reagents will not only increase the separation cost but also cause environmental pollution.

### 3.3. Enzymatic method

Enzymatic methods have the highest recovery rate of ESM after shell membrane separation, mainly using various enzymes to break the peptide bonds between the fibrous connections of ESM and the connections between the outer eggshell membrane and the mineralized layer of eggshell. Li (14) used alkaline protease and papain to separate and recover ESM, and conducted a comparative analysis with physical and chemical methods. The results showed that the highest amount of ESM was recovered through the enzymatic method, reaching 98.9% when using different enzymatic solutions for shell membrane separation. At the same time, configuring the enzymatic solution and the conditions of use become more and more important.

In summary, the physical method of shell membrane separation has the lowest recovery of ESM, but the cost is cheaper. The chemical method has medium efficiency but is faced with problems of membrane protein denaturation and waste liquid disposal. The enzymatic method has the highest efficiency of eggshell membrane recovery and can reduce membrane protein denaturation, but at a higher cost.

## 4. Advances in eggshell membrane proteolysis technology

Li (43) found in the eggshell membrane dissolution test that the eggshell membrane morphology did not swell in response to the lysis solution, suggesting the structure of the eggshell membrane was highly cross-linked. During the gradual dissolution and thinning of the eggshell membrane, the cystine content of the eggshell membrane decreased significantly while the content of other amino acids did not change significantly, suggesting the dissolution process of the eggshell membrane was accompanied by degrading cystine. It also indicated that the cleavage of disulfide bonds likely occurred during eggshell membrane dissolution. Further, the high insolubility of eggshell membrane proteins may be caused by inter- and intra-molecular disulfide bonds of the proteins (44) as well as the intercross-linking of ESM with lysine-derived latchins and iso-latchins (43). Therefore, reducing the extreme insolubility of ESM by cleaving the disulfide bonds is an important research direction to improve the solubility of ESM. Disulfide bonds are present in many kinds of proteins and peptides and are important covalent bonds to maintain protein stability. Studies have shown that disulfide bonds are formed by the oxidation of two sulfhydryl groups, so the protein insolubility caused by disulfide bonds can be reduced by converting disulfide bonds to sulfhydryl groups through redox reactions.

Methods for solubilization of eggshell membrane proteins can also be divided into mechanical type, combined chemistry type and adjuvant type, and a combination of these methods may yield better results. A typical eggshell membrane dissolution technique is shown in Table 3.

**Table 3:** Statistics of typical eggshell membrane dissolution techniques.

**Type**	**Dissolution characteristics**	**Membrane dissolution rate**	**References**
Mechanical method	Molecular changes caused by to act ultrasound energy	No change about the product	(45)
	Changes in the structure of proteins caused by to act ultrasound energy	Facilitates to extract lipids and proteins	(46)
Combined chemistry method	Alkaline condition treatment at 140°C	a large molecular weight protein	(47)
	Centrifugation of eggshell membrane powder mixed with 1% SDS solution	45%	(48)
	BCA	Suitable for protein identification	(49)
	3-Mercaptopropionic acid	62%	(3)
	DDT	Mixed extraction with high efficiency	(15)
Adjuvant method	Percarboxylic acid (10% hydrogen peroxide and 90% formic acid) and pepsin	39.30%	(44)
	5% papain, 100 mM sodium metabisulfite, PH is 6.2, dissolution time 12 h.	Obtain soluble eggshell membrane solution	(50)

### 4.1. Mechanical method

The main physical extraction methods of proteins include heating, pressurization and extrusion (2). The heating method generally refers to using a temperature between 100 and 200°C and the pressurization method generally uses pressures between 0.294 and 0.298 MPa. The increase in temperature increases the efficiency of the molecular thermal movement of the solution acting on the eggshell membrane and the increase in volume of solution facilitates the increase in contact area, which increases the eggshell membrane protein recovery (51). The extrusion method involves the crushing of solid raw materials in water and reducing additives to form a liquid phase, which is heated and melted in an extrusion device at a high temperature and pressure. All of these methods can degrade the disulfide bond due to β-elimination. It has been shown that the pressurization method can degrade the disulfide bonds of keratin up to 50–60%, and that heating destroys the spatial structure of the protein (52). Therefore, it is hypothesized that the disulfide bonds and spatial structure within the eggshell membrane proteins can also be disrupted by heating and pressurization, making the membrane proteins into easily soluble polypeptide compounds. This method may be significant for the extraction of specific eggs such as keratin but needs to be further explored for the extraction of other membrane proteins. In addition, Marcet (46) showed that changes in molecules and changes in the structure of proteins can be induced by the action of ultrasound energy, facilitating the extraction of lipids and proteins and improving the extraction of ESM proteins without changing the final product. Physical methods are rarely used for the extraction of eggshell membrane proteins at present due to their single action and low efficiency of membrane protein extraction.

### 4.2. Combined chemistry method

The main chemical methods for solubilizing eggshell membrane proteins are oxidation, reduction, and acid–alkali combination.

#### 4.2.1. Oxidation method

The oxidation method increases eggshell membrane solubility primarily by oxidizing the disulfide bonds between the fibers to sulfonic acid groups, thereby exposing more soluble fibrin to the solvent (53). The more effective oxidizing agent currently available is sodium dodecyl sulfate (SDS). Kritsda (47) centrifuged a mixture of eggshell membrane powder and 1% SDS solution and precipitated the supernatant membrane protein with acetone. The protein concentration was determined by the BCA (a sodium salt that binds highly specific to Cu^+^ to produce a purple complex) method (48) using bovine serum albumin (BSA) as a standard, and finally 45% protein extraction. The concentration of the SDS solution chosen for the extraction that was more suitable for protein identification did not result in the highest solubility rate for eggshell membrane proteins, suggesting that there may be room for further enhancement of the SDS solution solubility method.

#### 4.2.2. Reduction method

Reduction is a method to reduce the insolubility of ESM by breaking the disulfide bonds in the membrane through reducing reagents. The commonly used reducing reagents are mainly sulfhydryl compounds, which can open the disulfide bonds by exchange reactions with the disulfide bonds without breaking the peptide chains of proteins. Feng (3) treated eggshell membrane powder with 1.25 mol/L 3-mercaptopropionic acid and 10% acetic acid at 80°C for 20.5 h. The final membrane protein solubility reached 62% (eggshell membrane solubility is the ratio of the dry weight of the solid obtained from the dissolved protein solution after freeze concentration to the dry weight of the eggshell membrane before dissolution). In addition, Hincke (3) used a mixture of tris (2-carboxyethyl) phosphine hydrochloride buffer (TCEP-HCl), tris-hydroxymethyl aminomethane hydrochloride (Tris-HCl), and dithiothreitol (DTT) to solubilize proteins from ESM, resulting in a final eggshell membrane solubilization rate of 53% and 472 proteins. This method uses anothers the reducing properties of TCEP-HCl, which has a trialkylphosphine group that smoothly and quantitatively reduces organic disulfides to thiols in water, thereby breaking the disulfide bond. 3-mercaptopropionic acid and TCEP-HCl both reduce the disulfide bond, but their reducing properties differ, with TCEP-HCl having a higher theoretical reduction than 3-mercaptopropionic acid. However, in the reported eggshell membrane solubilization results (54), suitable ratios of 3-mercaptopropionic acid obtained better solubilization, implying that there is room for further improvement of the TCEP-HCl solubilization method.

#### 4.2.3. Acid–alkali method

Acid–alkali methods can be divided into acid, base, and acid–alkali combination methods. Acid (33) methods can use inorganic acids such as hydrochloric acid and acetic acid because disulfide bonds are more stable under acidic conditions and can be used with reducing reagents such as mercaptopropionic acid to extract eggshell membrane proteins. The alkali method usually uses a strong alkali solution such as NaOH to break disulfide bonds or even peptide bonds to obtain a lower molecular weight protein solution. Wang (55) used an acid method (hydrochloric acid) and an alkali method (sodium hydroxide) to extract keratin from pig hair and compared their results. It was found that the hydrolysis of pig hair by alkali and acid methods was weakened in order. The acid–alkali method is a combination of HCl and NaOH. HCl is a strong acid and treating the eggshell membrane can swell some of the proteins and hydrolyze them at a certain temperature, however, this also breaks the disulfide and peptide bonds within the eggshell membrane. Operationally, ESM are usually treated with HCl first to increase the solubility of membrane proteins, followed by adjusting the pH of the solution with NaOH to precipitate proteins with different isoelectric points. However, due to the strong acid property of HCl, the disulfide bonds in the membrane were broken by this method, while other parts of the proteins were denatured, which affected the identification of eggshell membrane proteins. For example, Chaitanya (56) have extracted keratin by the acid–alkali combination method.

### 4.3. Adjuvant method

The use of enzymes to solubilize ESM does not result in violent reactions, and the process is easily regulated by temperature control, but there is also the problem of incomplete reactions. This method is commonly used to solubilize specific proteins. For example, in 1996 Takahashi (44) used percarboxylic acid (10% hydrogen peroxide and 90% formic acid) to treat ESM at 25°C for 24 h, followed by vacuum filtration of distilled water for washing, and finally solubilization in pepsin and 0.5 mol/L acetic acid (25°C) for 24 h. The recovery of SEP obtained was approximately 39.3%. In addition Lee (57) presumed that the soluble protein solubility of ESM could reach up to 91.4% by orthogonal test using acetic acid and pepsin in acetic acid–enzyme coupled with segmental extraction technique. The solubilization of ESM by enzyme preparation method can retain the three-stranded helix structure of proteins to the maximum extent and can also select different proteases for solubilization according to different experimental purposes. At the same time, it can also maximize the recycling of useful materials and fully exploit the application value of ESM. However, enzyme preparation requires high reaction conditions, and the experimental application is difficult.

In summary, the physical method is simple, but the dissolution rate of eggshell membrane proteins is low. The enzymatic method is highly controllable, environmentally friendly, and mild, but the protein dissolution rate is also relatively low, and the protein species obtained is singular. The chemical method is vigorous and not easy to control, but it can open the disulfide bond better and can obtain higher membrane dissolution rates and eggshell membrane abundance. Currently, the treatment of eggshell membrane with reducing reagents or oxidizing reagents is more commonly used.

## 5. Conclusion

The shell membrane separation of eggshells and the sorting and utilization of eggshells and ESM are conducive to exploiting the potential utilization value of eggshells and turning them into treasure, reducing the waste of resources, and greatly protecting the environment. The physical, chemical, and enzymatic methods described in the article have produced good results for eggshell membrane separation. Each method has its own advantages and disadvantages, thus it is necessary to further broaden our knowledge of eggshell membrane separation, improve the effect of shell membrane separation, and further utilize eggshell resources.

ESM provide a mineralized platform for eggshell formation and have great potential for application due to their specific physical structure and chemical composition, but their insolubility has been a constraint to their research and exploitation. The presence of disulfide bonds within the eggshell membrane and the complex membrane fiber structure are the most important factors limiting the solubility of ESM according to existing studies. In the reported studies, the solubilization methods of ESM focused on the disulfide bond disruption in the internal structure, and the best solubilization effect was achieved by using sulfhydryl compounds with 62% solubilization rate. To improve the solubilization rate of ESM, it is recommended to optimize the experimental procedure of eggshell membrane solubilization based on existing studies, or to find chemical reagents and experimental methods that are more efficient in the conversion cleavage of disulfide bonds in ESM and less destructive to eggshell membrane proteins. Further analysis of the eggshell membrane microstructure may provide new perspectives for finding the cause of high protein insolubility and improving the eggshell membrane solubility rate.

## Author contributions

Design and complete the experiment: CH, LL, HC, and LS. Statistics and contributions: CH, DW, DZ, and YC. Provide experimental guidance: DW and ZN. All authors contributed to the article and approved the submitted version.
