# Corrigendum: Advances in eggshell membrane separation and solubilization technologies

**DOI:** 10.3389/fvets.2023.1216172

**Published:** 2023-05-17

**Authors:** Chunhao Han, Yifan Chen, Lei Shi, Hui Chen, Lanhui Li, Zhonghua Ning, Dan Zeng, Dehe Wang

**Affiliations:** ^1^College of Animal Science and Technology, Hebei Agricultural University, Baoding, China; ^2^College of Animal Science and Technology, China Agricultural University, Beijing, China; ^3^Hebei Layer Industry Technology Research Institute, Handan, China

**Keywords:** chicken, eggshell membrane, protein, disulfide bond, dissolution

**Error in Figure Legend**

In the published article, there was an error in the legend for Figure 1 as published. Reference (18) should have been cited as the image source. The corrected legend appears below.

**Figure 1:**
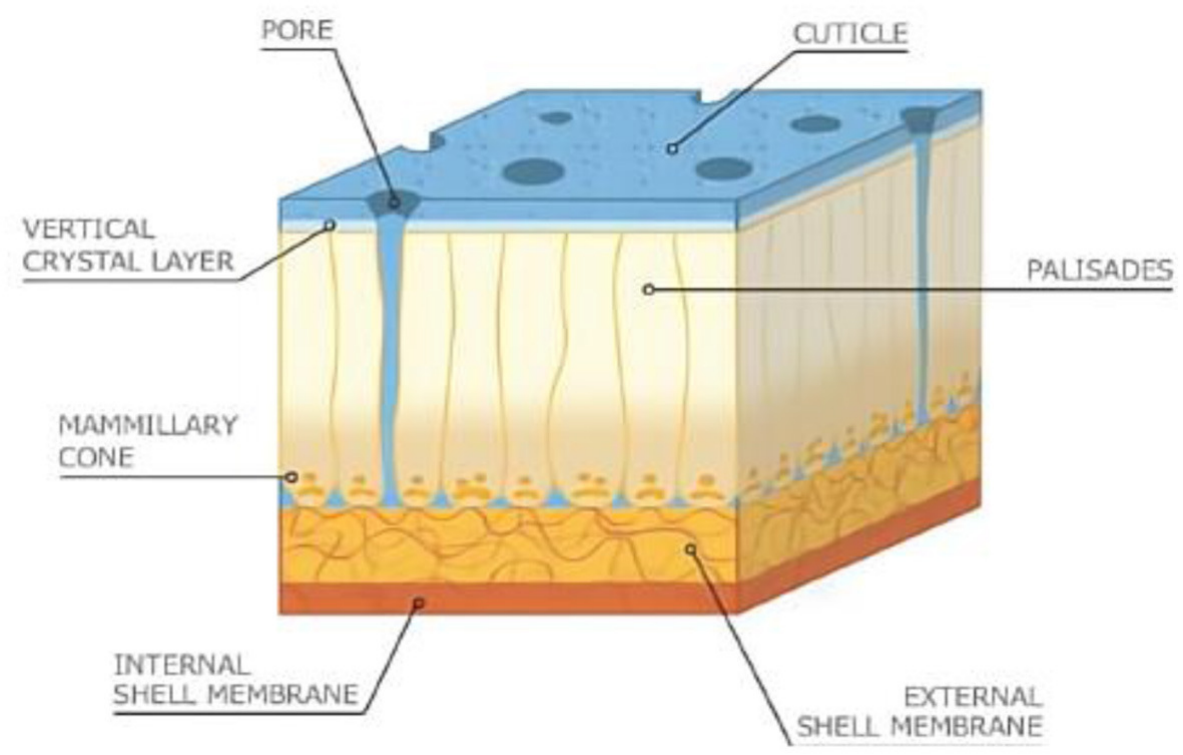
Artistic rendition of cross-sectional view of eggshell. Image reproduced from Hincke et al. (18).

**Incorrect Reference**

In the published article, the reference for (18) was incorrectly written as “Goldberg M, Kulkarni AB, Young M, Boskey A. The eggshell: structure, composition and mineralization. *Front Bioscie*. 3:711–35. doi: 10.2741/e281”.

It should be “Hincke MT, Nys Y, Gautron J, Mann K, Rodriguez-Navarro AB, McKee MD. The eggshell: Structure, composition and mineralization. *Front Biosci (Landmark Ed)*. (2012) 17:1266–80. doi: 10.2741/3985”

The authors apologize for these errors and state that this does not change the scientific conclusions of the article in any way. The original article has been updated.
